# EZH2 inhibition promotes epithelial-to-mesenchymal transition in ovarian cancer cells

**DOI:** 10.18632/oncotarget.11497

**Published:** 2016-08-22

**Authors:** Horacio Cardenas, Janice Zhao, Edyta Vieth, Kenneth P. Nephew, Daniela Matei

**Affiliations:** ^1^ Northwestern University Feinberg School of Medicine, Department of Obstetrics and Gynecology, Chicago, IL, USA; ^2^ Oakland University William Beaumont School of Medicine, Rochester, MI, USA; ^3^ Indiana University Department of Medicine, Indianapolis, IN, USA; ^4^ Medical Sciences, Indiana University, Bloomington, IN, USA; ^5^ Northwestern University Feinberg School of Medicine, Department of Medicine, Chicago, IL, USA; ^6^ Robert Lurie Cancer Center, Northwestern University, Chicago, IL, USA

**Keywords:** ovarian cancer, EZH2, EMT, H3K27me3, GSK126

## Abstract

Cancer cells acquire essential characteristics for metastatic dissemination through the process of epithelial-to-mesenchymal transition (EMT), which is regulated by gene expression and chromatin remodeling changes. The enhancer of zeste homolog 2 (EZH2), the catalytic subunit of the polycomb repressive complex 2 (PRC2), catalyzes trimethylation of lysine 27 of histone H3 (H3K27me3) to repress gene transcription. Here we report the functional roles of EZH2-catalyzed H3K27me3 during EMT in ovarian cancer (OC) cells. TGF-β-induced EMT in SKOV3 OC cells was associated with decreased levels of EZH2 and H3K27me3 (*P*<0.05). These effects were delayed (~72 h relative to EMT initiation) and coincided with increased (>15-fold) expression of EMT-associated transcription factors *ZEB2* and *SNAI2*. EZH2 knockdown (using siRNA) or enzymatic inhibition (by GSK126) induced EMT-like changes in OC cells. The EMT regulator *ZEB2* was upregulated in cells treated with either approach. Furthermore, TGF-β enhanced expression of *ZEB2* in *EZH2* siRNA- or GSK126-treated cells (*P*<0.01), suggesting that H3K27me3 plays a role in TGF-β-stimulated *ZEB2* induction. Chromatin immunoprecipitation assays confirmed that TGF-β treatment decreased binding of EZH2 and H3K27me3 to the *ZEB2* promoter (*P*<0.05). In all, these results demonstrate that EZH2, by repressing *ZEB2,* is required for the maintenance of an epithelial phenotype in OC cells.

## INTRODUCTION

The Polycomb group protein complexes alter methylation of histone tails and play a critical role during development by compacting chromatin and repressing transcription of differentiation-associated genes. Among the best studied chromatin regulators is enhancer of zeste homolog 2 (EZH2), the catalytic subunit of the polycomb repressor complex 2 (PRC2). EZH2 contains a Suvar 3-9 Enhancer of zeste Trithorax (SET) domain and functions as a histone methyltransferase (HMT) for the well characterized repressive mark histone H3 lysine 27 trimethylation (H3K27me3) [1]. Deregulation and function-altering mutations of chromatin modifiers [2-4] have been widely reported in cancer, including in ovarian cancer (OC), but their involvement and regulation in tumorigenesis are complex and remain not fully understood [5].

The concept that the epigenome may buffer DNA from environmental insults is an area of emerging interest. In cancer cells, environmental stimuli have been shown to induce changes in chromatin [6], but the mechanisms underlying chromatin remodeling in response to environmental cues is unclear. One of the key regulatory factors in the tumor microenvironment is the transforming growth factor β1 (TGF-β), which has been widely implicated in cancer progression [7]. TGF-β exerts both tumor suppressor and oncogenic activities in cancer [8] and while its effects are mediated transcriptionally, the cytokine has recently been shown to alter chromatin organization [9]. In this study, we sought to determine whether chromatin modifications imparted by EZH2 play a role in the response of OC cells to TGF-β.

In OC, TGF-β is abundantly secreted in the peritoneal environment by cancer and stromal cells [10, 11] and induces cancer cell invasiveness, epithelial to mesenchymal transition (EMT), and peritoneal metastasis [8, 12, 13]. We and others have shown that TGF-β signaling is active in OC [11, 12] inducing EMT, which permits break-down of cell junctions and loss of cell polarity allowing epithelial cells to become migratory and invasive [14]. While the genetic events triggered by the cytokine, such as activation of the E-cadherin repressors, *Twist, ZEB1/2* and *SNAI1/2* are well delineated [15], it is not clear whether TGF-β has additional effects on chromatin conformation and if such changes contribute to gene regulation linked to the EMT program.

To understand whether the cellular response to TGF-β involves epigenetic alterations, we recently completed a methylome analysis and demonstrated that TGF-β induced changes in DNA methylation that directly affected gene transcription [16]. DNA methylation is strongly correlated with polycomb repression [17, 18], prompting us to further dissect how the PRC2 complex responds to TGF-β and whether the EMT process is regulated by removal of repressive histone marks. By using a TGF-β-inducible EMT model in OC cells, we show that EZH2 is required for maintaining the epithelial characteristics of cancer cells and that erasure of the H3K27me3 marks catalyzed by EZH2 permits transition to a mesenchymal state. We also report that *ZEB2,* an important regulator of the late phases of EMT, is directly repressed by H3K27me3 and that removal of this transcriptional break is required for sustaining the mesenchymal phenotype induced by TGF-β. In all, our data support that EZH2 functions as repressor of a key EMT inducer preserving an epithelial phenotype and that TGF-β-induced gene regulation involves not only a well-defined molecular signaling pathway, but also chromatin modifications that appear to be necessary during the later phases of EMT enabling the maintenance of a mesenchymal phenotype.

## RESULTS

### Kinetics of TGF-β-induced EMT in OC cells

**Figure 1:**
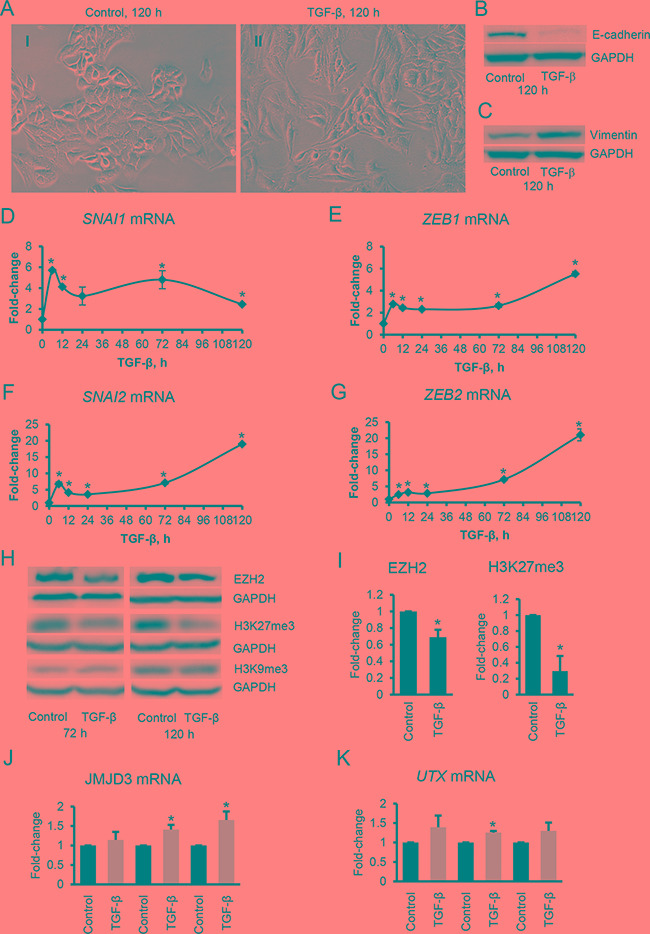
TGF-β induces EMT and changes in EZH2 and H3K27me3 in ovarian cancer cells **A.** Phase contrast microscopy identifies morphological changes induced in SKOV3 cells by TGF-β (5 ng/mL) at 120 hours (magnification: 200X). **B, C.** Western blotting for E-cadherin (B) and vimentin (C) in SKOV3 cells treated with TGF-β (5 ng/mL) for 120 hours. **D-G.** Relative changes in mRNA expression levels of the EMT inducers *SNAI1*, *SNAI2*, *ZEB1* and *ZEB2* in SKOV3 cells treated with TGF-β (5 ng/mL) for 6, 12, 24, 72, and 120 hours. mRNA expression levels were measured by qRT-PCR and normalized to GAPDH. Values are means ± SE (n=3). *Indicate significant difference (*P*<0.05) relative to control. **H.** Western blotting for EZH2, H3K27me3, and H3K9me3 in SKOV3 cells treated with TGF-β (5 ng/mL) for 72 and 120 hours. **I.** Quantification of EZH2 (n=5) and H3K27me3 (n=3) levels in SKOV3 cells treated with TGF-β (5 ng/mL) for 120 hours. Intensity of protein bands measured by densitometry was normalized to GAPDH levels. Bars represent means ± SE, *indicate *P*<0.05 relative to control. **J, K.** qRT-PCR measures mRNA expression levels of JMJD3 and UTX in SKOV3 cells treated with TGF-β (5 ng/mL) for the indicated times. Expression levels were normalized to GAPDH. Bars represent means ± SE (n=3). *Denote significant differences relative to control (*P*<0.05).

TGF-β-induced EMT [16] in SKOV3 cells was confirmed by examining changes in cellular shape from epithelial to a mesenchymal morphology (Figure 1A-1I, -1II), a decrease in protein levels of E-cadherin (Figure 1B), and an increase of expression levels of the mesenchymal marker, vimentin (Figure 1C). The morphological changes became evident at 24-48 hours and progressed during TGF-β stimulation up to 120 hours. Downregulation of E-cadherin (*CDH1* gene) during EMT is induced by a well described transcriptional machinery including the repressors *SNAI1,* *SNAI2* (Slug)*, TWIST, ZEB1, ZEB2,* and *E47* [19-23]. To understand the dynamic involvement of these transcription factors in TGF-β-induced EMT in OC cells, we studied their expression levels in a time course experiment. We observed an increase in *mRNA* levels of *SNAI1, SNAI2, ZEB1* and *ZEB2* at 12 hours, and a slight decrease and plateau from 48-120 hours. In contrast, *mRNA* levels of *SNAI2* and *ZEB2* were significantly increased (approximately 20-fold) at later time-points (120 h) (Figure 1D-1G). These data suggest that in this model, several transcription factors including *SNAI1, SNAI2, ZEB1* and *ZEB2,* initiate EMT*,* while maintenance of the mesenchymal phenotype depends mostly on *SNAI2* and *ZEB2*.

### Histone H3 trimethylation during TGF-β-induced EMT in OC cells

Because of observed differences in timing of transcriptional factor engagement by TGF-β, we hypothesized that the delayed overexpression of *SNAI2* and *ZEB2* could be regulated through epigenetic mechanisms [24]. To determine whether TGF-β stimulation is associated with changes in histone methylation, we measured the total levels of the H3K27me3 by western blotting. H3K27me3 levels decreased significantly 72 hours after TGF-β treatment and remained repressed at 120 hours (Figure 1H and 1I). This was accompanied by a modest decrease in protein levels of the methyltransferase EZH2 (Figure 1H and 1I). In contrast, levels of H3K9me3, another repressive mark not mediated by EZH2, were not altered by stimulation with TGF-β (Figure 1H). Interestingly, TGF-β did not alter EZH2 protein levels in untransformed ovarian surface epithelial (IOSE) cells (Supplementary Figure S1) suggesting that effects may be restricted to transformed cells. Additionally, TGF-β stimulation had no effect on EZH2 *mRNA* levels in SKOV3 cells (Supplementary Figure S2).

Because the H3K27me3 levels are influenced by both H3K27 methylation as well as by demethylation, we also examined the expression of the specific H3K27 demethylases *UTX* (also known as *KDM6A*) and *JMJD3* (also known as *KDM6B*). Stimulation with TGF-β increased (*P*<0.05) the *mRNA* expression levels of *JMJD3* at 72 and 120 hours (Figure 1K). TGF-β also increased (*P*<0.05) *UTX* expression (72 h) but to a lesser extent than *JMJD3* (Figure 1J). These results support that TGF-β induces a decrease in the total amount of the repressive mark H3K27me3 by decreasing EZH2 protein level and by increasing expression of H3K27 demethylases.

### Inhibition of EZH2 enzymatic activity induces mesenchymal characteristics in OC cells

**Figure 2:**
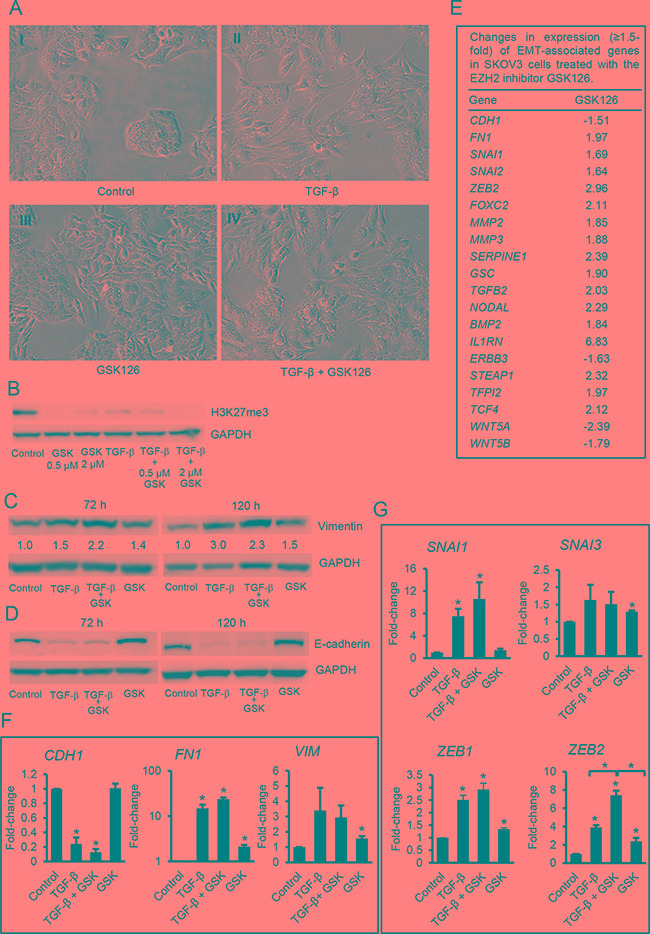
Effects of GSK126 on ovarian cancer cells **A.** Phase contrast microscopy identifies morphological changes induced in SKOV3 cells by diluent (control, A-I), TGF-β (5 ng/mL, picture A-II), GSK126 (2 μM, picture A-III), or the combination (picture A-IV) for 120 hours (magnification: 200X). **B.** Western blotting for H3K27me3 in SKOV3 cells treated with TGF-β (5 ng/mL), GSK126 (2 μM or 0.5 μM), or the combination for 120 hours. **C, D.** Effects of TGF-β (5 ng/mL), GSK126, and the combination on Vimentin (C) and E-cadherin (D) protein expression levels as measured by western blotting at 72 and 120 hours. Densitometric values relative to GADH are marked. **E.** List of EMT-associated genes and fold-change (≥ 1.5) in response to treatment with GSK126 (2 μM for 120 hours) measured by RT^2^ Profiler PCR arrays for EMT. mRNA expression levels were normalized to the average expression of five housekeeping genes (*ACTB, B2M, GAPDH, HPRT1 and RPLP0*). **F, G.** Validation using qRT-PCR of mRNA expression levels for (F) EMT markers *CDH1* (E-cadherin), *FN1* (fibronectin-1), and *VIM* (vimentin), and (G) EMT-associated transcription factors *SNAI1*, *SNAI3*, *ZEB1*, and *ZEB2* in SKOV3 cells treated with TGF-β (5 ng/mL), GSK126 (2 μM), or the combination for 120 hours. GAPDH was used for normalization. Bars represent means ± SE (n=3). *Indicate significant differences compared to controls or between values linked by brackets (*P*<0.05).

To determine whether the process of EMT involves events regulated by H3K27me3, we used the enzymatic inhibitor GSK126 and siRNA-mediated knock down. GSK126 is a specific S-adenosyl-methionine-competitive inhibitor of the EZH2 methyltransferase activity [25]. SKOV3 cells treated with GSK126 alone for 120 hours exhibited a change in shape from cobblestone to a mesenchymal morphology (Figure 2A-2I vs -2III). The morphological changes induced by GSK126 were similar to those induced by TGF-β (Figure 2A-2II). The combination of TGF-β plus GSK126 potently induced mesenchymal characteristics (Figure 2A-2IV). H3K27me3 levels were decreased by GSK126, TGF-β, or the combination treatment (Figure 2B), and EZH2 protein levels were decreased by TGF-β and were not affected by GSK126 (Supplementary Figure S3), in agreement with previous reports [25]. The mesenchymal morphology induced by GSK126 and by TGF-β correlated with an increased expression of vimentin (Figure 2C) and N-cadherin (Supplementary Figure S4). Interestingly however, the levels of E-cadherin did not change after GSK126 treatment (Figure 2D), suggesting that the enzymatic activity of EZH2 is not implicated in the regulation of E-cadherin expression.

To further explore the involvement of EZH2 and H3K27me3 during the process of EMT, we used an RT-PCR array to determine changes in expression of 84 known EMT-associated genes in SKOV3 cells treated with 2 μM GSK126 for 120 hours. Twenty genes showed ≥1.5-fold change in cells treated with GSK126 including EMT inducers and mesenchymal markers (Figure 2E). Quantitative RT-PCR subsequently confirmed that EZH2 inhibition significantly increased (*P*<0.05) expression levels of the mesenchymal markers *FN1* (fibronectin 1) and *VIM* (vimentin) (Figure 2F) and the EMT inducers *SNAI3*, *ZEB1,* and *ZEB2* (Figure 2G). In most cases, the levels of up-regulation induced by GSK126 were less than those induced by TGF-β. However, the upregulation of *ZEB2* induced by TFG-β and by GSK126 were comparable, and the combination of TGF-β and GSK126 had an additive effect (*P*<0.01) on *ZEB2* expression compared to each agent alone (Figure 2G).

**Figure 3:**
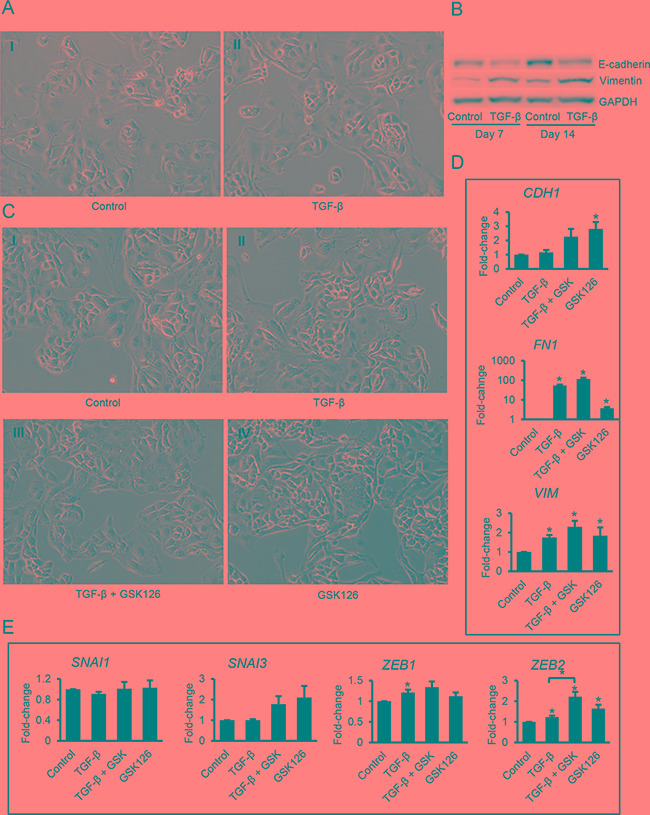
Effects of TGF-β and GSK126 on Kuramochi ovarian cancer cells **A.** Phase contrast microscopy identifies morphological changes induced in Kuramochi cells by TGF-β (5 ng/ml) at 7 days (magnification: 200X). **B.** Western blotting for E-cadherin and vimentin in lysates of Kuramochi cells treated with TGF-β (5 ng/mL) for 7 and 14 days. **C.** Morphology of Kuramochi cells treated with vehicle (C-I), TGF-β (5 ng/mL, C-II), TGF-β (5 ng/mL) plus GSK126 (2 μM, C-III), or GSK126 (2 μM, C-IV) for 7 days (magnification: 200X). **D, E.** Relative mRNA expression levels (means ± SE, n=3) of *CDH1* (E-cadherin), *FN1* (fibronectin) and *VIN* (Vimentin) (D) and EMT-associated transcription factors *SNAI1*, *SNAI3*, *ZEB1*, and *ZEB2* (E) measured by qRT-PCR in Kuramochi cells treated as described in C for 96 hours. mRNA expression levels were normalized to GAPDH. Bars represent means ± SE. *Indicate significant differences relative to control or between bars linked by brackets (*P*<0.05).

Next, we examined the induction of EMT in a different OC cell line. EMT was induced in Kuramochi cells by TGF-β after prolonged treatment (up to 14 days) as indicated by subtle changes in morphology to elongated shapes (Figure 3A-3I vs 3A-3II), a decrease of E-cadherin protein levels and an increase in vimentin expression (Figure 3B). Kuramochi cells treated with GSK126, or TGF-β, alone or in combination for 96 hours, showed a similar mesenchymal-like morphology (Figure 3C). GSK126 treatment increased (*P*<0.05) expression of the mesenchymal markers *FN1* and *VIM* (Figure 3D) and EMT inducer *ZEB2* (Figure 3E). The effect of combining TGF-β plus GSK126 on *ZEB2* expression was greater (*P*=0.01) than TGF-β alone (Figure 3E), similar to the responses observed in SKOV3 cells.

### EZH2 knockdown induces a mesenchymal phenotype in SKOV3 cells

**Figure 4:**
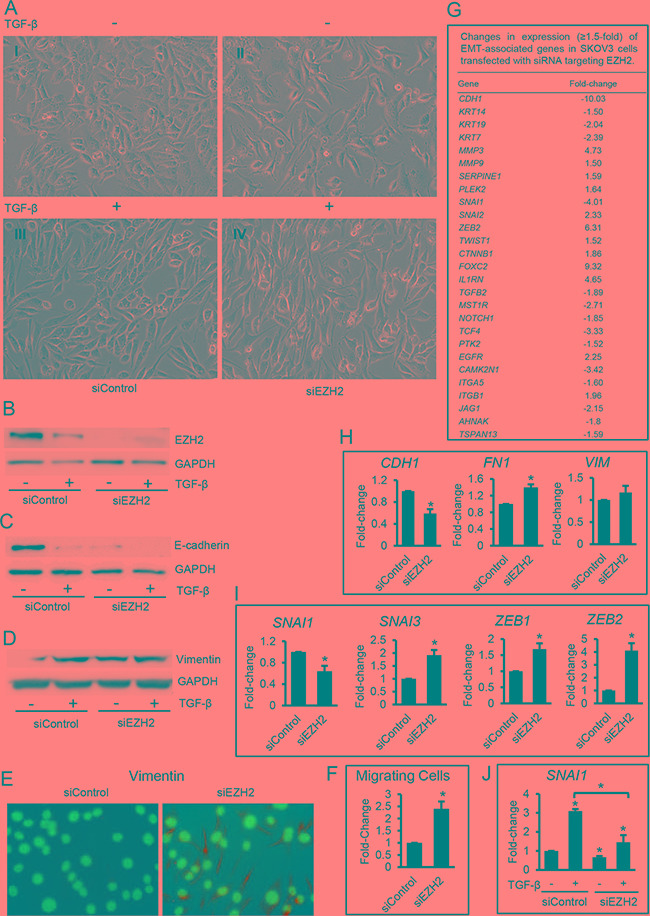
Effects of *EZH2* knock down by siRNA in ovarian cancer cells **A.** Morphology of SKOV3 cells transfected with control siRNA (siControl, A-I and A-III) or siRNA targeting *EZH2* (siEZH2, A-II and A-IV), and treated with control vehicle (AI and II) or TGF-β (5 ng/mL-AII and IV) for 48 hours (magnification: 200X). **B, C, D.** Western blotting of EZH2 (B), E-cadherin (C), and vimentin (D) in SKOV3 cell lysates treated as described in A. **E.** Immunofluorescent staining of vimentin (green) in SKOV3 cells transfected with control siRNA (siControl) or siRNA targeting *EZH2* (siEZH2) (magnification: 400X). Nuclei show blue DAPI staining. **F.** Transwell migration of SKOV3 cells treated as described in E. **G.** List of EMT-associated genes and fold-change (≥1.5) in SKOV3 cells transfected with siRNA targeting *EZH2* or control siRNA, as measured by RT^2^ Profiler PCR arrays for EMT. mRNA expression levels were normalized to the average expression of five housekeeping genes (*ACTB, B2M, GAPDH, HPRT1 and RPLP0*). **H, I.** Validation using qRT-PCR of mRNA expression levels for (H) EMT markers *CDH1* (E-cadherin), *FN1* (fibronectin-1), and *VIM* (vimentin), and (I) EMT-associated transcription factors *SNAI1*, *SNAI3*, *ZEB1*, and *ZEB2* in SKOV3 cells transfected with siRNA targeting *EZH2* (siEZH2) or control siRNA (siControl). GAPDH was used for normalization. **J.** qRT-PCR measurements of *SNAI1* mRNA expression levels in SKOV3 cells transfected with control siRNA (siControl) or siRNA targeting *EZH2* (siEZH2) and then treated with vehicle or TGF-β (5 ng/mL) for 48 hours. mRNA expression levels were normalized to GAPDH. Bars represent means ± SE (n=3). *Indicate significant differences compared to controls or between bracket-linked means (*P*<0.05).

To further examine the effects of EZH2 inhibition on EMT we used siRNA targeting EZH2 (siEZH2). Western blot confirmed efficient EZH2 knockdown by siEZH2 compared with scrambled siRNA (siControl, Figure 4B). SKOV3 cells transfected with siControl and treated with TGF-β displayed a mesenchymal morphology indicative of EMT (Figure 4A-4I vs. -4III). SKOV3 cells transfected with siEZH2 and either treated or untreated with TGF-β also exhibited a mesenchymal morphology (Figures 4A-4II and 4A-4IV), a decrease in protein levels of E-cadherin (Figure 4C), an increase in vimentin (Figure 4D and 4E), and an increase in migratory properties (transwell migration, Figure 4F) consistent with induction of EMT.

The expression of EMT-associated genes in SKOV3 cells transfected with siRNA targeting EZH2 was next examined by using a quantitative RT-PCR EMT array. Interestingly, more EMT associated genes were affected by EZH2 knockdown compared to enzymatic inhibition. Specifically, EZH2 knockdown caused an increase in *mRNA* expression levels for the EMT inducers *SNAI2*, *ZEB2*, *TWIST1*, and of genes with known roles in mesenchymal cell function (*MMP3*, *MMP9*, *SERPINE1*, and *PLEK2),* and a decrease of the epithelial markers *CDH1*, *KRT14*, *KRT19*, and *KRT7* (Figure 4G). The effects of EZH2 knockdown on EMT induction were confirmed by qRT-PCR which showed a decrease in *CDH1* (Figure 4H), and increases in *FN1* (Figure 4H) and the EMT inducers *ZEB1*, *ZEB2,* and *SNAI3*, (Figure 4I). Taken together with the previous data obtained using the enzymatic inhibitor GSK126, these results support that inhibition of EZH2 induces EMT in OC cells. Consequently, relatively high EZH2 expression levels (and likely activity) appear necessary for maintaining an epithelial phenotype.

Interestingly, the only transcriptional regulator linked to EMT, which was suppressed, rather than upregulated by EZH2 knockdown was *SNAI1* (Figure 4I). It is possible that *SNAI1* is not necessary for EMT induction in this model and that its functions are redundant with those of other TGF-β-inducible transcription factors. This is consistent with results observed with the use of GSK126 which induced mesenchymal cell characteristics without causing any changes in *SNAI1* expression (Figure 2G and 3E). Repression of *SNAI1* *mRNA* levels induced by EZH2 knockdown could also indicate that EZH2 directly regulates its expression. To examine this possibility, we determined the effect of EZH2 knockdown on TGF-β-induced *SNAI1* upregulation in SKOV3 cells. Indeed, we observed TGF-β-induced upregulation of *SNAI1* in cells expressing normal levels of EZH2 but no upregulation in cells transfected with siEZH2 and treated with TGF-β (Figure 4J), indicating that EZH2 is required for TGF-β-induced *SNAI1* upregulation.

### TGF-β decreases EZH2 binding to and H3K27me3 marking of the ZEB2 promoter

**Figure 5:**
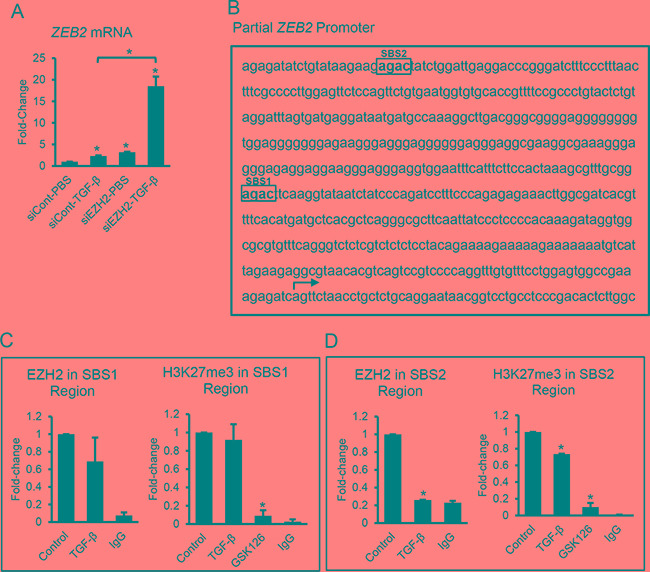
TGF-β and EZH2 regulation of *ZEB2* expression **A.** qRT-PCR measures *ZEB2* mRNA expression levels (means ± SE, n=3) in SKOV3 cells transfected with control siRNA (siControl) or siRNA targeting *EZH2* (siEZH2) and treated with vehicle (control) or TGF-β for 48 hours. mRNA expression levels were normalized to GAPDH. Bars represent means ± SE (n=3). *Indicate significant differences compared to controls or between bracket-linked values (*P*<0.05). **B.** Partial sequence of the *ZEB2* promoter illustrates two SMAD binding sites (designated as SBS1 and SBS2). The transcription start site (TSS) is marked by an arrow. **C, D.** Chromosome immunoprecipitation measures effects of TGF-β on EZH2 and H3K27me3 binding to the *ZEB2* promoter containing the SBS1 (C) and SBS2 (D). GSK126 decreases H3K27me3 binding to the same regions (positive control). Input levels were used for normalization. Bars represent means ± SE (n=2). *Indicate significant differences relative to control (*P*<0.05).

Among the EMT inducers, *ZEB2* which appears to be important to the late stages of EMT (Figure 1G) was the most responsive based on fold-change to EZH2 inhibition by GSK126 (Figure 2F) or knockdown (Figure 4I). To test its involvement relative to EZH2 in TGF-β-induced EMT, SKOV3 cells transfected with control or siRNA targeting EZH2 were treated with TGF-β for 48 hours. TGF-β notably upregulated (~17-fold) *ZEB2* expression in SKOV3 cells transfected with siEZH2 relative to cells transfected with control siRNA (*P*<0.05, Figure 5A). These results are consistent with the *ZEB2* response observed in cells treated with TGF-β and the EZH2 inhibitor GSK126 (Figure 2F), suggesting that the *ZEB2* promoter may be marked by the H3K27me3 repressive mark, and could become more responsive to TGF-β upon removal of the histone mark. To determine whether TGF-β alters EZH2 binding to and H3K27me3 marking of the *ZEB2* promoter, we used ChIP with antibodies against EZH2 and H3K27me3. We examined within the -700 to +300 sequence relative to the transcription start site (TSS) of the *ZEB2* promoter, two regions containing SMAD binding sites (SBS, minimal consensus sequence AGAC) designated SBS1 (-103 to -256) and SBS2 (-428 to -585) (Figure 5B). Pull down of the region containing the SBS1 using EZH2 and H3K27me3 antibodies was not affected by TGF-β (Figure 5B). However, TGF-β decreased (*P*<0.05) EZH2 binding and the amount of H3K27me3 in the region containing the SBS2 (Figure 5D). As a control, SKOV3 cells treated with the EZH2 inhibitor GSK126 displayed decreased (*P*<0.05) levels of H3K27me3 marking in the two examined regions (Figure 5C and 5D). These results demonstrate that TGF-β decreases H3K27me3 repressive marking in specific regions of the *ZEB2* promoter, which may enable more efficient binding of TGF-β induced SMADs (or other transcription factors) that, in turn, regulate *ZEB2* expression.

### Inhibition of H3K27 demethylases suppresses TGF-B-induced EMT

**Figure 6:**
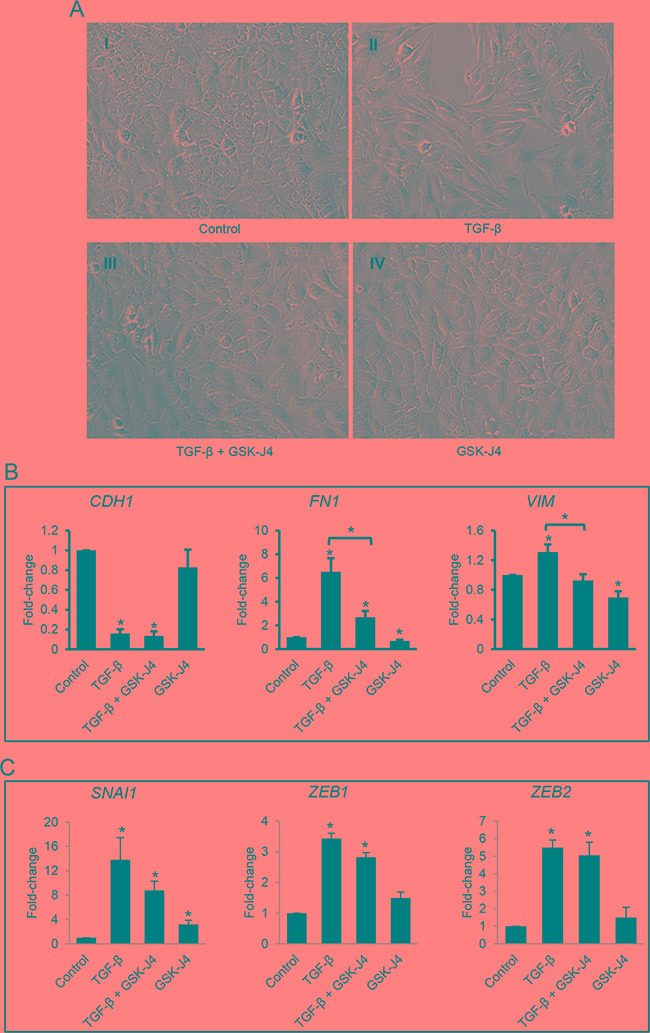
Effects of the JMJD3 and UTX inhibitor GSK-J4 on TGF-β-induced EMT in ovarian cancer cells **A.** Morphology accessed by phase contrast microscopy of SKOV3 cells treated with vehicle (A-I), TGF-β (5 ng/mL, A-II), TGF-β (5 ng/mL) plus GSK-J4 (4 μM, A-III), or GSK-J4 (4 μM, panel A-IV) for 96 hours (magnification: 200X). **B, C.** qRT-PCR measurements of mRNA expression levels for (B) EMT markers *CDH1*, *FN1*, and *VIM*, and (C) EMT-associated transcription factors *SNAI1*, *ZEB1*, and *ZEB2* in SKOV3 cells treated as described in A. GAPDH was used for normalization. Values are means ± SE (n=3). *Indicate significant differences relative to control or between bracket-linked values (*P*<0.05).

Stability of the H3K27me3 chromatin repressive marking depends on both the activity of the HMT EZH2 and of the H3K27 demethylases JMJD3 and UTX [26]. To examine the involvement of the demethylases JMJD3 and UTX, which we found to be modestly upregulated by TGF-β (Figure 1J and 1K) during the process of EMT, we used the small molecule inhibitor GSK-J4 which is enzymatically converted into GSK-J1 upon entering the cytosol and blocks both JMJD3 and UTX [27]. We observed that SKOV3 cells treated with GSK-J4 for 96 hours maintained an epithelial cobblestone appearance (Figure 6A-6IV) similar to that of control-treated cells (Figure 6A-6I). The mesenchymal phenotype induced by TGF-β (Figure 6A-6II) was prevented by concomitant treatment with GSK-J4 (Figure 6A-6III). GSK-J4 also inhibited (*P*<0.05) TGF-β-induced upregulation of the mesenchymal markers, *FN1* (fibronectin) and *VIM* (vimentin, Figure 6B), but did not alter the effects of TGF-β on *CDH1* (Figure 6B). These data add to the concept that H3K27me3 mediated repression of mesenchymal characteristics is responsive to TGF-β treatment. In contrast, the epithelial marker E-cadherin appears less affected by modulation of this histone mark in response to TGF-β. Furthermore, the E-cadherin repressors *SNAI1*, *ZEB1*, and *ZEB2,* which are potently induced by TGF-β, were only modestly affected by GSK-J4 (only *SNAI1*) and the demethylase inhibitor did not significantly alter their response to TGF-β (Figure 6C). In all, these results support that inhibition of JMJD3 and UTX by GSK-J4 partially suppresses TGF-β-induced EMT by preventing upregulation of mesenchymal markers.

## DISCUSSION

Epithelial-to-mesenchymal transition is a cellular process involved in the early phases of development and in cancer metastasis. Recent data support that regulation of this “plastic” process involves not only genetic factors, but also epigenetic remodeling, including alterations in DNA methylation and effects of histone modifiers on chromatin assembly [16, 28]. The Polycomb (PcG) and the Trithorax groups (TrxG) of proteins function as epigenetic repressors or activators respectively, being implicated in regulation of gene transcription during development [29]. It has been established that their loss of function impacts the fate of embryonic stem cells and their differentiation into the three germ layers [30]. In the present study we describe a novel mechanism by which EMT is sustained in OC cells through loss of the repressive chromatin mark H3K27me3 at the promoter of transcription factors involved in the induction of mesenchymal characteristics. Our data point to *ZEB2* as a transcriptional regulator activated late during the transition, whose promoter loses the H3K27me3 histone mark in response to TGF-β. Mesenchymal characteristics of OC cells are enhanced by both EZH2 knockdown or by enzymatic inhibitors, suggesting that EZH2 is required for maintaining the epithelial cellular characteristics in this system.

Consistent with our findings, a recent report described the methyltransferase activity of EZH2 as a regulator of mesenchymal to epithelial transition (MET) during human pluripotent cell reprogramming. In this model, EZH2 inhibitors prevented Oct4/Sox2/Klf4/c-Myc induced transformation of fibroblasts into iPSCs [31]. Likewise, it has been shown that inhibition of EZH2 augments the expression of transgelin/smooth muscle-22α, a mesenchymal marker, in response to TGF-β in endothelial cells [32]. Loss of EZH2 in the lung epithelium resulted in defective epithelial layer differentiation and alveoli formation [33]. Lastly, conditional overexpression of EZH2 using the MMTV-LTR model in the mammary gland caused epithelial hyperplasia through activation of the Wnt/β-catenin pathway [34], while conditional EZH2 knock-out delayed the outgrowth of the mammary epithelium [35]. In all, like our data, these studies support that EZH2 functions as a guardian of epithelial cell fate and perhaps, as a break against TGF-β-induced transition to mesenchymal features.

The increased expression of EZH2 in ovarian tumors [36] could explain the reported relative resistance of OC cells to EMT [37, 38], or their preference towards MET, reverting to an epithelial phenotype immediately after dissemination from the primary tumor site. The transition between EMT and MET may be particularly relevant to OC, where dissemination occurs intraperitoneally and cells are required to adopt an epithelial phenotype and to adhere to the mesothelium shortly after relocating to distant sites. It is possible that EZH2 facilitates this process during tumor metastasis, tilting the balance in favor of MET.

On the other hand, the findings that EZH2 enhances the epithelial characteristics of OC cells argue against the concept that this HMT is an oncogene associated with cancer metastasis [36, 39]. EZH2 was previously shown to repress E-cadherin in breast, pancreatic and prostate cancer cells [40, 41]. Cooperation between Snail1 and EZH2 in the repression of E-cadherin was also observed during neural crest development [42]. In this regard, our findings are discrepant, as we show that E-cadherin expression is suppressed by EZH2 knockdown in OC cells. Interestingly, we also observed that the EZH2 inhibitor GSK126 did not alter E-cadherin expression, suggesting that in our model, E-cadherin regulation by EZH2 is independent of its HMTase function. Polycomb-independent regulatory functions of EZH2 have been also recognized in other cancer models, where the protein cooperates with other transcription factors (such as the androgen receptor or NF-κB) to exert oncogenic properties [43, 44]. Our findings likely reflect EZH2's distinct cell context-dependent functions, which have been previously recognized [43]. Indeed, other studies have proposed a tumor suppressor role for EZH2, as loss-of-function mutations associated with decreased H3K27me3 levels were noted in patients with myelodysplastic syndromes and myeloproliferative neoplasms (MDS/MPN) [45, 46], and deletion of EZH2 was sufficient to induce MDS/MPN-like disease in mice [47].

We attribute the EMT blocking role of EZH2 to its repressive effects on several transcriptional regulators, and in particular on *ZEB2*. This is the first report demonstrating that *ZEB2* is a direct EZH2 target whose promoter, which is repressed by the H3K27me3 mark, responds to TGF-β stimulation. We show that blockade of EZH2 by using either siRNA mediated knockdown or pharmacological inhibitors leads to de-repression of *ZEB2* and drives OC cells through EMT. Polycomb-mediated regulation of the *ZEB1* promoter has been recently implicated in the conversion of breast cancer non-stem to stem cells [48] and loss of H3K27me3 at the poised promoter of *ZEB1* was functionally linked to activation of the stemness regulatory transcriptional machinery.

Our results also show that TGF-β stimulation is associated with a decrease of the H3K27me3 repressive mark, which appears to occur late during the EMT process. Consistent with our findings, Ramadoss, et al. [49] have shown a decrease of the H3K27me3 mark in response to TGF-β in a mammary epithelial system, which was attributed to upregulation of the demethylase JMJD3. We also observed in our OC cell model upregulated demethylase levels in response to TGF-β. However, the decrease of the repressive chromatin mark in our system appears to be related to both increased levels of demethylases as well as decreased EZH2 activity. Epigenetic reprogramming including a global reduction of the H3K9me2 marked heterochromatin involving LOCKS regions and an increase in H3K4Me3 associated euchromatin was described in hepatocytes exposed to TGF-β [9]. In monocytes conditioned by TGF-β, global mapping of histone marks showed that a larger number of differentiation associated genes was marked by H3K4me3, while significantly fewer genes were marked by H3K27me3 in response to the cytokine [50]. Collectively these data support that epigenomic modifications occur during EMT, are most likely cell context-dependent, and participate in the fine tuning of cellular transformation and plasticity with its acquired new functions.

Although EZH2 has been considered a potential cancer therapeutic target [51, 52], our findings also point to potentially unexpected consequences of EZH2 inhibition leading to de-repression not only of tumor suppressor genes that can block cancer growth, but also of pro-tumorigenic factors which can unleash unanticipated aggressive cancer behavior. The difficulty manipulating epigenomically-directed therapies is linked to their “imprecise targeting” and ability to globally reprogram the oncogenic machinery. We posit that an in-depth analysis of both potential benefits as well as adverse consequences must be *a priori* completed and understood. Efforts to design pathway specific epigenomic blocking strategies that avoid non-specific off-target effects are critically needed.

## MATERIALS AND METHODS

### Cell culture and treatments

SKOV3 cells were obtained from the American Type Culture Collection (ATCC). Kuramochi cells were from the Japanese Cancer Research Resources Bank. The immortalized ovarian surface epithelial (IOSE) cells were kindly provided by Dr. N. Auersperg from the University of British Columbia. SKOV3 and IOSE cells were maintained in media containing 1:1 MCDB 105 (Sigma-Aldrich, St. Louis, MO) and M199 (Cellgro, Tewksbury, MA). Kuramochi cells were cultured in RPMI 1640. Media were supplemented with 10% fetal bovine serum and 1% penicillin-streptomycin solution. Cells were treated with TGF-β1 (hereafter referred to as TGF-β, R&D Systems, Minneapolis, MN), EZH2 inhibitor GSK126 (Xcess Biosciences, Inc., San Diego, CA), UTX and JMJD3 inhibitor GSK-J4 (Sigma-Aldrich), or combinations of TGF-β with inhibitors at doses and durations as indicated.

### Cell transfection

Transient knockdown of *EZH2* was performed by transfecting SKOV3 cells with a pool of 3 target-specific small interfering RNA (siRNA) (catalogue#: sc-35312, Santa Cruz Biotechnology, Inc. Dallas, TX) in the presence of the DreamFect Gold transfection reagent (OZ Biosciences, San Diego, CA). Scrambled siRNA (Santa Cruz Biotechnologies, Inc.) served as control.

### Migration assay

Cell migration was evaluated using transwell inserts (8 μm pore size) in a 24-well plate (Corning Inc., Corning NY). Ten thousand cells suspended in serum-free medium were seeded in the upper compartment, and medium containing 20% FBS was added to the lower compartment as attractant. Cells were cultured for 24 hours under standard conditions. Non-migrating cells were removed with a cotton swab, and remaining migrating cells were fixed with 4% paraformaldehyde, stained with 1% crystal violet, and counted using a light microscope.

### RNA isolation and real-time quantitative RT-PCR (qRT-PCR)

Total RNA was extracted with the RNA Stat-60 reagent (Tel-Test, Inc., Friendswood, TX). An iScript cDNA Synthesis kit (Bio-Rad Laboratories, Hercules CA) was used for reverse transcription of RNA, and iTaq Universal SYBR Green Supermix (Bio-Rad) was used for quantitative PCR (qPCR) amplification of cDNAs following procedures from the manufacturers. Relative changes in mRNA expression levels were calculated by the 2^-ΔΔCT^ method using GAPDH for normalization. The sequences of primers used for qRT-PCR are presented in Supplementary Table S1. The human EMT RT^2^ Profiler PCR Array was purchased from SA Bioscience (Frederick, MD) and used with an ABI Prism 7900 HT Real-Time PCR system (Applied Biosystems, Foster City, CA), according to the manufacturer's instructions. Array data analysis was performed based on the 2^-ΔΔCT^ method with normalization of the raw data to the housekeeping genes included in the array using a Microsoft Excel algorithm provided by the manufacturer. Fold-changes were calculated for each gene in treated cells compared to controls. All experiments were repeated at least three times in independent conditions.

### Western blotting

Proteins were resolved by SDS-PAGE and electroblotted onto polyvinylidene difluoride membranes (Millipore, Billerica, MA). Membranes were blocked and then probed with antibodies against the proteins of interest. Antibodies against vimentin (# 3932), E-cadherin (#3195), EZH2 (#3147), and tri-methyl-histone H3 (Lys27) (H3K27me3, #9756) were from Cell Signaling Technology, tri-methyl-histone H3 (Lys9) (H3K9me3, ab8898) from Abcam Inc. (Cambridge, MA), and GAPDH (#H86504M) from Meridian Life Sciences, Inc. (Memphis, TN). Membranes were incubated with HRP-conjugated secondary antibody, followed by detection of antigen-antibody complexes using an enhanced chemiluminescent substrate (SuperSignal West Pico, Thermo Scientific, Waltham, MA). Digital images of blots were captured by a luminescent image analyzer (ImageQuant LAS 4000, GE Healthcare Life Sciences, Piscataway, NJ). Densitometric analysis of protein bands was performed with ImageJ 1.48 software (National Institutes of Health of the USA). Experiments were repeated at least three times.

### Immunofluorescence

Cells grown in 8-well culture slides (Millicell EZ slide, Millipore) were fixed with 4% paraformaldehyde for 30 min, and blocked/permeabilized with PBS containing 5% normal goat serum and 0.3% Triton X-100 for 60 minutes. Thereafter, cells were incubated with primary antibody overnight at 4°C, and then with Alexa Fluor 488-labeled anti-rabbit IgG (H+L) for 2 hours. Slides were coverslipped using Vectashield mounting medium with DAPI (Vector Laboratories, Burlingame, CA) and subsequently examined and photographed with a fluorescence microscope fitted with a digital camera.

### Chromosome immunoprecipitation (ChIP)

ChIP assays were performed using a kit (#17-295, Millipore) following the manufacturer's protocol. Briefly, chromatin was cross-linked with 1% formaldehyde for 10 minutes at 37°C. Cells were treated with SDS lysis buffer followed by sonication (5 pulses of 10 seconds each at 30 seconds interval using Fisher Scientific Model 100 set at 3). Protein-chromatin complexes were immunoprecipitated with antibodies against EZH2 (#5246, Cell Signaling Technology, Danvers, MA), H3K27me3 (#9756, Cell Signaling Technology), or IgG (negative control). Complexes were separated by incubation with salmon sperm DNA/protein A agarose slurry, and then the chromatin was eluted and the cross-links reversed. DNA was purified with a Chromatin IP DNA Purification kit (Active Motif, Carlsbad, CA). The primers used for amplification of specific regions of the *ZEB2* promoter by qPCR are described in Supplementary Table S2. Chromosome immunoprecipitation experiments were repeated at least twice.

### Statistical analysis

Data were analyzed using the Student's *t*-test. Differences were considered statistically significant when *P*<0.05.

## SUPPLEMENTARY MATERIALS FIGURES AND TABLES


