# Supplementary material for: EZH2 inhibition promotes epithelial-to-mesenchymal transition in ovarian cancer cells
Source: Oncotarget. 2016 Aug 22;7(51):84453–67. doi: 10.18632/oncotarget.11497 (PMC5356672; doi:10.18632/oncotarget.11497)
Supplement: Supplementary file 1 [file oncotarget-07-84453-s001.pdf]

EZH2 Inhibition promotes epithelial-to-mesenchymal transition in ovarian cancer cells

SUPPLEMENTARY FIGURES AND TABLES

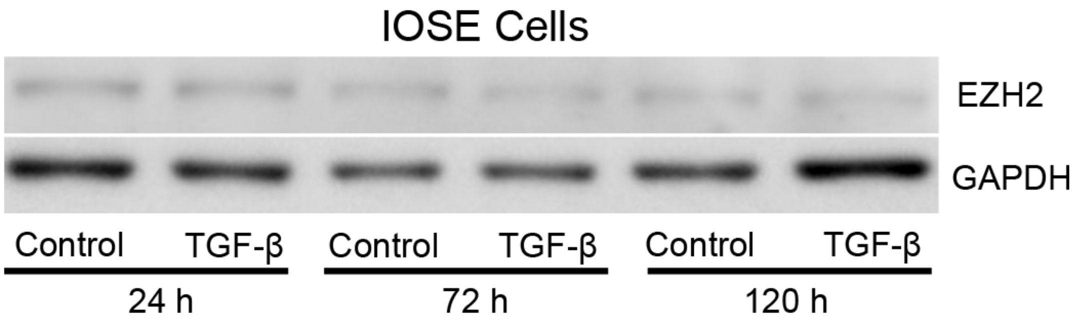

Supplementary Figure S1: Effects of TGF-β on EZH2 protein levels in IOSE cells. Cells were treated with TGF-β (5 ng/mL) for 24, 72 and 120 hours and EZH2 was measured by western blotting.

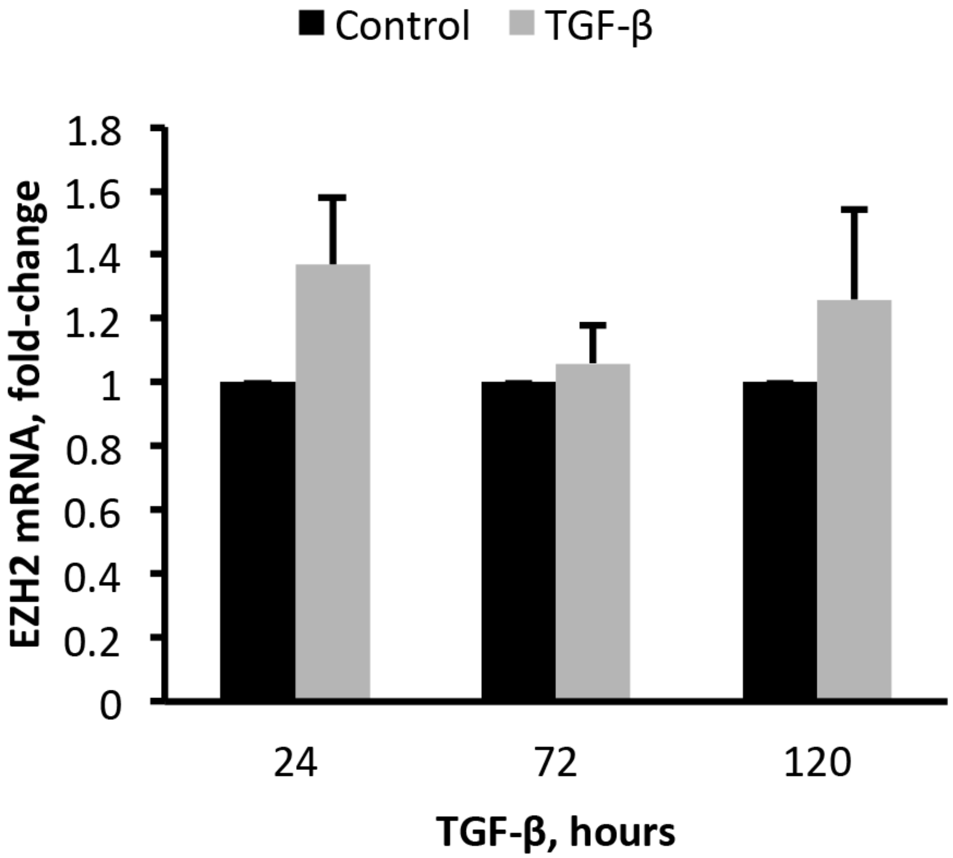

Supplementary Figure S2: *EZH2* mRNA levels in SKOV3 cells treated with TGF-β. SKOV3 cells were treated with TGF-β (5 ng/mL) for the periods indicated and *EZH2* was measured by real-time RT-PCR. Bars represent means ± SE (n=4).

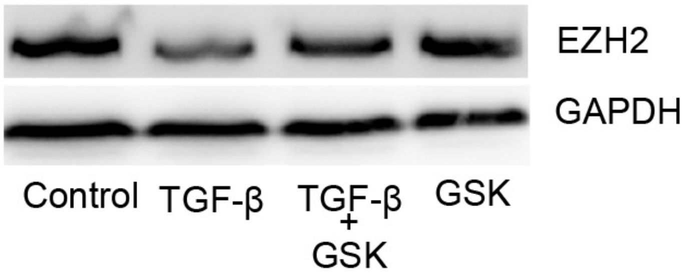

Supplementary Figure S3: Western blot of EZH2 in SKOV3 cells treated with TGF-β, EZH2 inhibitor GSK126, or combination for 120 hours.

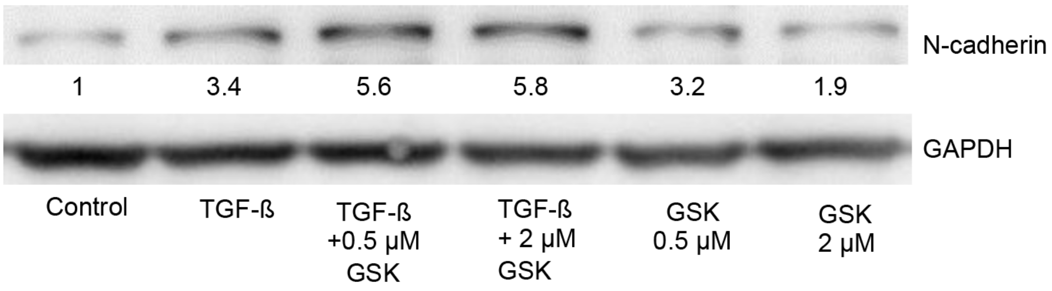

Supplementary Figure S4: Western blot of N-cadherin in SKOV3 cells treated with TGF-β, EZH2 inhibitor GSK126, or combinations for 120 hours. Numbers between bands are ratios of band intensities to GAPDH determined by densitometric analysis.

Supplementary Table S1: Sequences of the primers used for qRT-PCR

Gene	Sequences (5' to 3')
CDH1	Forward: GCCGAGAGCTACACGTTCA
	Reverse: GACCGGTGCAATCTTCAAA
FN1	Forward: AATATCTCGGTGCCATTTGC
	Reverse: CGGGAATCTTCTCTGTCAGC
VIM	Forward: GTTTCCCCTAAACCGCTAGG
	Reverse: AGCGAGAGTGGCAGAGGA
SNAI1	Forward: ACCCCACATCCTTCTCACTG
	Reverse: TACAAAAACCCACGCAGACA
SNAI2	Forward: TGGTTGCTTCAAGGACACAT
	Reverse: GTTGCAGTGAGGGCAAGAA
SNAI3	Forward: AAATCAATGGTGCCTGCTCT
	Reverse: GGCTGTCTTTGAGGGGTACA
ZEB1	Forward: AGGATGACCTGCCAACAGAC
	Reverse: TCTGCATCTGACTCGCATTC
ZEB2	Forward: CGCTTGACATCACTGAAGGA
	Reverse: CTTGCCACACTCTGTGCATT
KDM6A	Forward: AAGTGGAGGTTTTTGATCTG
	Reverse: CTTGGCAATGTACTATGTAGG
KDM6B	Forward: CAGGAGAATAACAACTTCTGC
	Reverse: CACAGGAATATTGGATGCATAG
GAPDH	Forward: AGCCACATCGCTCAGACAC
	Reverse: GCCCAATACGACCAAATCC

Supplementary Table S2: Sequences of the primers used for chromosome immunoprecipitation

Gene	Sequences (5' to 3')
ZEB2 Promoter (SBS1 region)	Forward: TAAAGCGTTTGCGGAGACTT
	Reverse: ACGAGAGACCCTGAAACACG
ZEB2 Promoter (SBS2 region)	Forward: GGGCAGAGAACTTTGTTCCA
	Reverse: GGTGCACACCATTCACAGAA
